# No causal associations between childhood family income and subsequent psychiatric disorders, substance misuse and violent crime arrests: a nationwide Finnish study of >650 000 individuals and their siblings

**DOI:** 10.1093/ije/dyab099

**Published:** 2021-05-29

**Authors:** Amir Sariaslan, Janne Mikkonen, Mikko Aaltonen, Heikki Hiilamo, Pekka Martikainen, Seena Fazel

**Affiliations:** 1 Social and Public Policy Unit, Faculty of Social Sciences, University of Helsinki, Helsinki, Finland; 2 Population Research Unit, Faculty of Social Sciences, University of Helsinki, Helsinki, Finland; 3 UEF Law School, University of Eastern Finland, Joensuu, Finland; 4 Finnish Institute for Health and Welfare (THL), Helsinki, Finland; 5 Centre for Health Equity Studies (CHESS), Stockholm University and Karolinska Institutet, Stockholm, Sweden; 6 Max Planck Institute for Demographic Research, Rostock, Germany; 7 Department of Psychiatry, University of Oxford, Warneford Hospital, Oxford, UK

**Keywords:** Socio-economic status, family income, schizophrenia, bipolar disorder, depression, anxiety, substance-use disorders, violence, quasi-experimental research designs, public health

## Abstract

**Background:**

Childhood family income has been shown to be associated with later psychiatric disorders, substance misuse and violent crime, but the consistency, strength and causal nature of these associations remain unclear.

**Methods:**

We conducted a nationwide cohort and co-sibling study of 650 680 individuals (426 886 siblings) born in Finland between 1986 and 1996 to re-examine these associations by accounting for unmeasured confounders shared between siblings. The participants were followed up from their 15^th^ birthday until they either migrated, died, met criteria for the outcome of interest or reached the end of the study period (31 December 2017 or 31 December 2018 for substance misuse). The associations were adjusted for sex, birth year and birth order, and expressed as adjusted hazard ratios (aHRs). The outcomes included a diagnosis of a severe mental illness (schizophrenia-spectrum disorders or bipolar disorder), depression and anxiety. Substance misuse (e.g. medication prescription, hospitalization or death due to a substance use disorder or arrest for drug-related crime) and violent crime arrests were also examined. Stratified Cox regression models accounted for unmeasured confounders shared between differentially exposed siblings.

**Results:**

For each $15 000 increase in family income at age 15 years, the risks of the outcomes were reduced by between 9% in severe mental illness (aHR = 0.91; 95% confidence interval: 0.90–0.92) and 23% in violent crime arrests (aHR = 0.77; 0.76–0.78). These associations were fully attenuated in the sibling-comparison models (aHR range: 0.99–1.00). Sensitivity analyses confirmed the latter findings.

**Conclusions:**

Associations between childhood family income and subsequent risks for psychiatric disorders, substance misuse and violent crime arrest were not consistent with a causal interpretation.

**Key Messages**

- The causal nature between childhood family income and subsequent risks for psychiatric disorders, substance misuse and violent crime remains unclear.
- In this Finnish cohort study of 650 680 individuals, we initially found that increased family income was associated with lower risks of psychiatric disorders, substance misuse and arrest for a violent crime.
- However, once we compared siblings who grew up in the same household but were exposed to varying income levels at specific ages, the associations were no longer present.
- Associations between family income and subsequent psychiatric disorders, substance misuse and violent crime arrest were therefore explained by shared familial risks and were not consistent with a causal interpretation.

## Introduction

Around a fifth of children in high-income countries are reported to live in relative income poverty, ranging from ∼10% in the Nordic countries to 30% in the USA.^1^ Children and adolescents who grow up in low-income households are typically at least twice as likely as their peers to develop schizophrenia-spectrum,^2^^,^^3^ mood and anxiety disorders,^4^ as well as to engage in substance misuse and violent crime.^5^^,^^6^ Furthermore, longitudinal studies have found that upward mobility in family income predicts better psychiatric outcomes in children than persistent income poverty.^3^^,^^4^^,^^6^ Expert opinion has postulated that these links between family income and later psychiatric morbidity are partly causal. Recommendations have been made that mental health professionals take an active role in advocating for reductions of population-level income differences and to implement interventions to assist low-income families in the direction of upward income mobility.^3^^,^^7–9^ However, the extent to which such actions can specifically reduce rates of psychiatric disorders and related behaviours partly depends on the observed associations being causal, which has yet to be rigorously tested.

A key methodological limitation of the literature is that alternative hypotheses, such as unmeasured familial confounding (i.e. that the associations are accounted for by omitted familial risk factors that cause low parental income and increased rates of offspring psychiatric disorders, substance misuse and violent crime), have been largely ignored.^10^ The importance of such mechanisms is underlined by twin studies that have consistently reported that income phenotypes are moderately to considerably heritable (∼40–60%).^11^ These findings are expected because heritable characteristics, such as cognitive abilities, impulsivity and personality traits, increasingly predict differences in social outcomes as societies move towards meritocratic systems.^12–14^ Moreover, there is considerable evidence that psychiatric disorders are also heritable (∼30–80%)^15–18^ and molecular genetic studies have recently estimated genetic correlations between income and many psychiatric and neurodevelopmental disorders^13^^,^^19^ Genetically informative research designs that can explicitly account for unmeasured familial risk factors and thereby isolate the residual, and potentially causal, effects of environmental risk markers (i.e. family income) are required to move the field beyond identifying associations and inform the design of potentially effective interventions.^10^

Therefore, we have conducted a study of the entire population of Finland born between 1986 and 1996 who resided in the country during their childhood and adolescence, including >650 000 individuals. We used high-quality nationwide registries to examine how family-income trajectories during childhood and adolescence were associated with subsequent risks for psychiatric disorders, substance misuse and violent crime arrest. We assessed the role of unmeasured familial confounders by comparing the rates of the outcomes between differentially exposed siblings who, due to having different birth years, were exposed to varying levels of family income at specific ages.

## Methods

We defined the target study population as individuals who met the following criteria: (i) born in Finland between 1 January 1986 and 31 December 1996, (ii) registered to reside in Finland on 31 December 2000 and (iii) could be linked to both biological parents (*n *=* *705 887). All Finnish residents are assigned a unique personal identification number, which is used in different nationwide registers and provides accurate linkage.^20^ We were granted permission to use anonymized data from Statistics Finland following an approval by their Ethics Board (TK-53–525-11). Informed consent is not a requirement for register-based studies in Finland.

Data on socio-demographic factors were retrieved from annual population registries covering the entire population of Finland between 1987 and 2018. The Causes of Death register, maintained by Statistics Finland, provided mortality dates in addition to their underlying and contributory causes (International Classification of Diseases, ICD-8, -9 and -10; 1969–2017; coverage >99%).^21^ Individuals diagnosed with psychiatric disorders and substance-use disorders were identified from the Care Register for Health Care, maintained by the Finnish Institute for Health and Welfare, which includes inpatient-care episodes (ICD-8, -9 and -10; 1969–2017) and specialist outpatient visits (ICD-10; 1998–2017) but not primary-care visits or care provided by smaller private clinics.^22^ The specialist outpatient-care data are routinely collected from all publicly funded secondary care providers, including hospitals and other specialist clinics.^22^ The Prescription Drug Register, maintained by the Social Insurance Institution of Finland, provided data on all prescription drug purchases in the Anatomical Therapeutic Chemical classification system (1995–2018). All criminal arrests recorded by the police authorities since 1996 were derived from registers maintained by Statistics Finland. We were restricted to annual arrest dates, which we set to 31 December for each year.

Our analytical sample included individuals who were in the national population registers from birth up to age 15 years and had at least one parent included in the national censuses during the same period (*n *=* *650 680), covering 92.2% of the targeted sample. Within the analytical sample, we identified 426 886 full-siblings in 185 327 families. The participants were followed up from their 15^th^ birthday until they either migrated, died, experienced the outcome of interest or were alive at the end of the follow-up period (31 December 2017 or 31 December 2018 for substance misuse).

The primary exposure was family income, defined as the sum of both parents’ inflation-adjusted gross taxable income in 2019 USD values, measured at the end of the year in which the offspring turned 15 years of age. These data were obtained from the tax authorities. Consistently with previous studies,^23^^,^^24^ we divided the family income measure by the square root of the number of family members to correct for differences in family size. We also considered alternative income measures, including disposable family income, either with or without family size corrections, in complementary sensitivity analyses.

We examined three categories of psychiatric disorders, including severe mental illness (e.g. schizophrenia-spectrum disorder and bipolar disorder), depression and anxiety (ICD-codes are presented in Supplementary Table S1, available as Supplementary data at *IJE* online). In addition, we examined substance misuse (e.g. a diagnosis of a substance use disorder, an arrest for a drug-related crime or a prescription for medications used in alcohol- and opioid-addiction treatment; definitions are presented in Supplementary Tables S2 and S3, available as Supplementary data at *IJE* online) and violent crime arrest. The validity of single-episode schizophrenia-spectrum disorder (positive predictive value, PPV: 75–100%),^25^ bipolar disorder (PPV = 87–93%),^25^ depression (PPV = 0.81)^26^ and alcoholic psychosis (PPV = 92%)^25^ diagnoses have been shown to range from fair to excellent in Finland.

### Analytical approach

First, we estimated adjusted hazard ratios (aHRs) by fitting a series of Cox regression models that examined the associations between family income at age 15 years and subsequent risks of the psychiatric disorders, substance misuse and violent crime arrest whilst accounting for the time at risk. We initially fitted separate models that gradually accounted for an increasing number of measured confounders. In the crude model, we only adjusted for sex, birth year and birth order. Second, we fitted an adjusted model that additionally accounted for immigrant background, urbanicity, single-parent household, parental educational attainment as well as specific parental psychiatric disorders (e.g. severe mental illness, depression and anxiety), substance misuse and violent crime arrest; definitions are presented in Supplementary Table S4, available as Supplementary data at *IJE* online. Third, we fitted a stratified Cox regression model, in which we allowed the baseline hazard rate to vary across clusters of siblings, which implies that the comparisons were made within nuclear families and between the siblings rather than between unrelated individuals. This approach allowed us to account for all unmeasured factors that were shared between the siblings, which included approximately half of their co-segregating genes and their shared childhood environmental influences. The degree to which the adjusted population estimates were attenuated within families indicated the level of unmeasured familial confounding. We explored timing and accumulation effects by rerunning the crude and sibling-comparison models for the annual family income measures at ages 1, 5 and 10 years in addition to the mean family income accumulated across all four measurements. We conducted visual inspections of scaled Schoenfeld residuals across time to assess whether the proportional-hazards assumption of the models was met.^27^ Multiple testing correction was performed using the Holm-Bonferroni method.^28^

In sensitivity analyses, we fitted group-based trajectory models,^4^^,^^29^ which allowed us to identify groups of individuals who were exposed to distinct family-income trajectories (Supplementary Material, available as Supplementary data at *IJE* online). We then compared risks of the outcomes between siblings who were exposed to different family-income trajectories. We also considered alternative exposures (e.g. parental receipt of means-tested social assistance, unemployment benefits and disability pension) and outcomes, including individual diagnoses of severe mental illnesses (e.g. schizophrenia, schizophrenia-spectrum disorders and bipolar disorder) and specific substance misuse outcomes (e.g. alcohol- and drug-use disorders as well as drug-related crime arrests). The coverage of the outpatient care data was not completed until 2006 due to differences in reporting practices between hospital districts.^22^^,^^30^ To test whether these differences affected our findings, we excluded all data on outpatient-care visits prior to 2006. Furthermore, we tested for the potential impact of reverse causation bias by excluding individuals who met criteria for any of the outcomes before their 15^th^ birthday (*n *=* *12 555). We also tested for within-family effect modification of family income by sex, birth year and birth order by including interaction terms in the sibling-comparison models. We explored potential non-linear within-family associations by refitting the sibling-comparison models and applying a series of natural cubic spline transformations of the family income exposure with up to 10 degrees of freedom.^31^ The natural cubic spline and interaction models were separately compared with the main sibling models to assess their fit.

The sibling-comparison design makes a number of assumptions, including siblings being generalizable to the population, exposures having sufficient within-family variability and the absence of sibling carry-over effects (e.g. the exposures and outcomes being uncorrelated between siblings). To test for these assumptions, we initially fitted the crude model on the sibling sample and compared the estimates with the crude model in the full sample. We then estimated sibling correlations for all of the family income measures using linear mixed-effects models,^32^ which allowed us to assess the relative proportion of the income differences that were either shared within families or unique to each sibling. To satisfy distributional assumptions, we log-transformed the income measures in these specific analyses. Lastly, we relaxed all three of these assumptions by running complementary analyses using differentially exposed cousins, who share on average 12.5% of their co-segregating genes. We identified 243 282 full-cousins who were nested within 63 396 extended families. To avoid comparing differentially exposed siblings nested within extended families, we decided to stratify the Cox-regression models across 651 652 cousin pairs who shared the same maternal-side grandmother but not the same mother. As individuals were nested within multiple cousin pairs, we used individual-specific cluster-robust standard errors in these analyses. To test for residual environmental confounding, we also examined cousin pairs who resided in different municipalities at age 15 years (*n*_cousins_ = 188 641; *n*_extended families_ = 48 091).

## Results

Socio-demographic characteristics and parental histories of psychiatric disorders, substance misuse and violent crime, stratified across those who met criteria for the outcomes (*n *=* *127 461 or 19.6%) and controls (*n *=* *523 219 or 80.4%), are presented in Table 1. Compared with individuals who did not meet criteria for any of the outcomes, we found that those with the examined outcomes were exposed to higher levels of psychosocial adversities (Table 1). The follow-up period was ∼11 years across the outcomes (range: 10.8–11.9) and the rates varied between 2.1 per 1000 person-years for severe mental illness to 8.0 per 1000 person-years for depression (Table 2).

**Table 1.** Socio-demographic characteristics of individuals born in Finland in 1986–1996 (*n* = 650 680)

	Controls	Severe mental illness	Depression	Anxiety	Substance misuse	Violent crime arrest
Birth order						
**Individuals**	522 195	15 530	56 000	39 710	39 611	44 578
**Median age at first diagnosis or event [years (IQR)]**	–	21.2 (18.7-24.1)	20.0 (17.3-23.2)	20.9 (18.1-23.9)	20.8 (18.8-23.5)	19.6 (17.6-22.2)
**Sex**						
Men	263 116 (50.4%)	7697 (49.6%)	19 334 (34.5%)	14 924 (37.6%)	27 310 (68.9%)	33 838 (75.9%)
Women	259 079 (49.6%)	7833 (50.4%)	36 666 (65.5%)	24 786 (62.4%)	12 301 (31.1%)	10 740 (24.1%)
**Birth year**						
1986	44 487 (8.5%)	1782 (11.5%)	5339 (9.5%)	3468 (8.7%)	3972 (10.0%)	5148 (11.5%)
1987	43 945 (8.4%)	1656 (10.7%)	5164 (9.2%)	3357 (8.5%)	3747 (9.5%)	4870 (10.9%)
1988	46 325 (8.9%)	1671 (10.8%)	5441 (9.7%)	3584 (9.0%)	3973 (10.0%)	4969 (11.1%)
1989	46 375 (8.9%)	1618 (10.4%)	5378 (9.6%)	3620 (9.1%)	3756 (9.5%)	4688 (10.5%)
1990	48 301 (9.2%)	1646 (10.6%)	5512 (9.8%)	3881 (9.8%)	3999 (10.1%)	4537 (10.2%)
1991	48 405 (9.3%)	1534 (9.9%)	5478 (9.8%)	3858 (9.7%)	3919 (9.9%)	4244 (9.5%)
1992	49 535 (9.5%)	1490 (9.6%)	5610 (10.0%)	4059 (10.2%)	3746 (9.5%)	4124 (9.3%)
1993	48 872 (9.4%)	1267 (8.2%)	5082 (9.1%)	3698 (9.3%)	3552 (9.0%)	3674 (8.2%)
1994	49 464 (9.5%)	1153 (7.4%)	4814 (8.6%)	3764 (9.5%)	3437 (8.7%)	3368 (7.6%)
1995	48 866 (9.4%)	978 (6.3%)	4363 (7.8%)	3310 (8.3%)	2925 (7.4%)	2690 (6.0%)
1996	47 620 (9.1%)	735 (4.7%)	3819 (6.8%)	3111 (7.8%)	2585 (6.5%)	2266 (5.1%)
First	217 643 (41.7%)	6462 (41.6%)	23 169 (41.4%)	16 816 (42.3%)	16 373 (41.3%)	17 953 (40.3%)
Second	179 675 (34.4%)	5130 (33.0%)	18 599 (33.2%)	13 110 (33.0%)	13 316 (33.6%)	14 624 (32.8%)
Third	81 254 (15.6%)	2499 (16.1%)	9000 (16.1%)	6301 (15.9%)	6499 (16.4%)	7491 (16.8%)
Fourth or higher	43 623 (8.4%)	1439 (9.3%)	5232 (9.3%)	3483 (8.8%)	3423 (8.6%)	4510 (10.1%)
**Immigrant background**	18 279 (3.5%)	639 (4.1%)	2233 (4.0%)	1567 (3.9%)	1865 (4.7%)	2053 (4.6%)
**Urbanicity**						
Urban	291 022 (55.7%)	9605 (61.8%)	33 879 (60.5%)	24 494 (61.7%)	24 600 (62.1%)	25 631 (57.5%)
Semi-urban	84 421 (16.2%)	2298 (14.8%)	8399 (15.0%)	5892 (14.8%)	5904 (14.9%)	7072 (15.9%)
Rural	146 752 (28.1%)	3627 (23.4%)	13 722 (24.5%)	9324 (23.5%)	9107 (23.0%)	11 875 (26.6%)
**Single-parent household**	29 235 (5.6%)	1463 (9.4%)	5179 (9.2%)	3632 (9.1%)	4618 (11.7%)	5056 (11.3%)
**Highest parental level of education**						
Primary	23 559 (4.5%)	1035 (6.7%)	3753 (6.7%)	2755 (6.9%)	3498 (8.8%)	4841 (10.9%)
Secondary	344 574 (66.0%)	10 326 (66.5%)	38 055 (68.0%)	26 765 (67.4%)	28 024 (70.7%)	33 257 (74.6%)
Tertiary	154 062 (29.5%)	4169 (26.8%)	14 192 (25.3%)	10 190 (25.7%)	8089 (20.4%)	6480 (14.5%)
**Parental psychiatric disorders and antisocial behaviors**						
Parental severe mental illness	22 168 (4.2%)	2106 (13.6%)	5271 (9.4%)	3711 (9.3%)	3424 (8.6%)	3428 (7.7%)
Parental depression	69 601 (13.3%)	4469 (28.8%)	15 866 (28.3%)	10 725 (27.0%)	10 089 (25.5%)	10 362 (23.2%)
Parental anxiety	35 892 (6.9%)	2163 (13.9%)	7440 (13.3%)	5743 (14.5%)	4887 (12.3%)	5225 (11.7%)
Parental substance misuse	57 968 (11.1%)	3407 (21.9%)	11 717 (20.9%)	8221 (20.7%)	10 389 (26.2%)	11 426 (25.6%)
Parental violent-crime arrest	51 340 (9.8%)	2751 (17.7%)	9715 (17.3%)	6766 (17.0%)	9134 (23.1%)	11 920 (26.7%)

**Table 2.** Person-time at risk, number of cases and rates per 1000 person-years among individuals born in Finland in 1986–1996 and followed up until 31 December 2017 (31 December 2018 for substance misuse)

	Person-time at risk (years)		Number of cases	Rate per 1000 person-years (95% CI)
	Total	Average per person (SD)		
Severe mental illness	7 280 833	11.2 (3.3)	15 530	2.1 (2.1–2.2)
Depression	7 024 231	10.8 (3.7)	56 000	8.0 (7.9–8.0)
Anxiety	7 158 842	11.0 (3.5)	39 710	5.5 (5.5–5.6)
Substance misuse	7 744 619	11.9 (3.6)	39 611	5.1 (5.1–5.2)
Violent crime arrest	7 060 102	10.9 (3.6)	44 578	6.3 (6.3–6.4)

**Figure 1.**
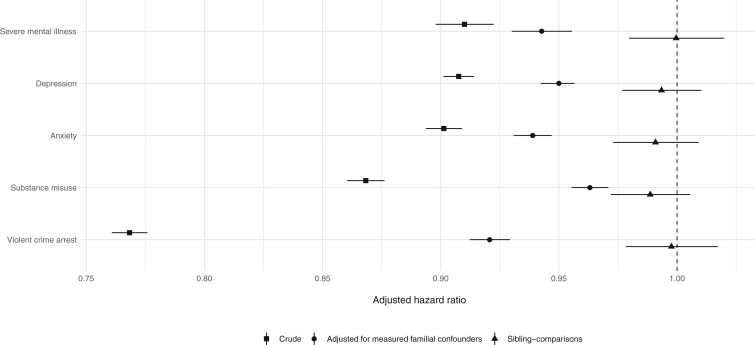
Associations between family income at age 15 years (in units of $15 000) and subsequent psychiatric disorders, substance misuse and violent-crime arrest among individuals born in Finland in 1986–1996 and followed up until 31 December 2017 (31 December 2018 for substance misuse). All models were adjusted for sex, birth year and birth order. The adjusted-for-measured-confounders models were adjusted for immigrant background, urbanicity, single-parent household, parental educational attainment, psychiatric disorders, substance misuse and violent-crime arrest.

We initially found that for each $15 000 increase in family income measured at age 15 years, risks of the outcomes were reduced by between 9% (adjusted hazard ratio, aHR = 0.91; 95% confidence interval: 0.90–0.92) for being diagnosed with a severe mental illness to 23% (aHR = 0.77; 0.76–0.78) for being arrested of a violent crime (Figure 1). Following adjustments for an extensive set of measured familial confounders, these associations remained but were attenuated (aHR range: 0.92–0.96; Figure 1). However, when we compared the risks of the outcomes between differentially exposed siblings to account for unmeasured familial confounders, we found that the associations were entirely attenuated (aHRs: 0.99–1.00; *P *>* *0.05; Figure 1). Consistently with the absence of non-linear effects, none of the other ways to measure family income improved the fit of the sibling models (*P *>* *0.05) and we did not find evidence for any within-family effect modification of family income by either birth order, birth year or sex (*P *>* *0.05). The findings remained intact when we excluded incomplete outpatient care data prior to 2006 (Supplementary Figure S1, available as Supplementary data at *IJE* online), individuals who met criteria for the outcomes before the baseline of the study (Supplementary Figure S2, available as Supplementary data at *IJE* online) and when we considered alternative outcome definitions, including psychotropic medication prescriptions (Supplementary Figure S3, available as Supplementary data at *IJE* online).

**Figure 2.**
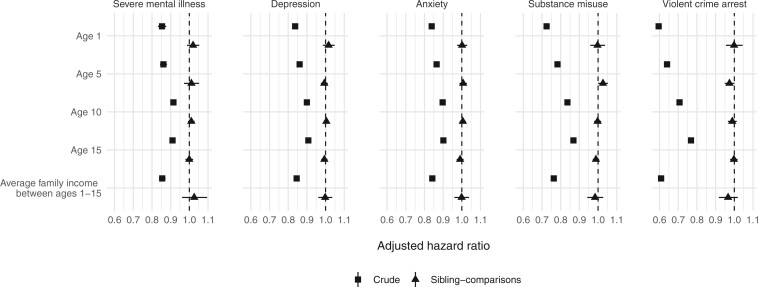
Associations between family income measured across ages 1–15 years (in units of $15 000) and subsequent psychiatric disorders, substance misuse and violent-crime arrest among individuals born in Finland in 1986–1996 and followed up until 31 December 2017 (31 December 2018 for substance misuse). All models were adjusted for sex, birth year and birth order. The average family-income measure was calculated using four measurement points (e.g. ages 1, 5, 10 and 15 years).

Although the strength of the crude population-wide associations varied temporally when we considered earlier measurements of family income at ages 1, 5 and 10 years (aHR_crude_ range 0.60–0.91; Figure 2) in addition to the mean family income accumulated across all four measurement points (aHR_crude_ range 0.61–0.85; Figure 2), all of the corresponding sibling-comparison estimates were fully attenuated (aHR range 0.97–1.03; *P *>* *0.05; Figure 2). We obtained similar results when we considered alternative definitions of family income (Supplementary Figure S4, available as Supplementary data at *IJE* online), including a categorical measure of family-income trajectories (Supplementary Material, available as Supplementary data at *IJE* online) and other indicators of parental socio-economic status (Supplementary Figure S5, available as Supplementary data at *IJE* online).

To assess the underlying assumptions of the sibling-comparison model, we carried our several sensitivity analyses. The siblings in our sample did not systematically differ from the full population as the crude associations were comparable across both subsets (Supplementary Figure S6, available as Supplementary data at *IJE* online). The null findings within families could not be attributed to insufficient income variability, as the sibling correlations for the family income exposures increased from 0.71 to 0.80 across the four measurement points, thus indicating that between ∼20% and 30% of the observed income differences were unique to siblings within families. Despite only accounting for 12.5% of unmeasured genetic confounders, we found that associations derived from comparing differentially exposed cousins were attenuated by a median of 40.3% (range: 28.0–44.4%; Supplementary Figure S7, available as Supplementary data at *IJE* online) relative to the crude population estimates, thus providing additional validity to the sibling-comparison analyses. We found negligible differences when restricting the analyses to cousin pairs who resided in different municipalities at age 15 years (Supplementary Figure S7, available as Supplementary data at *IJE* online).

## Discussion

In this nationwide cohort study of 650 680 individuals born in Finland between 1986 and 1996 and followed up until 2018, we found that the population-wide associations between family income during childhood and adolescence on subsequent risks of a wide range of psychiatric disorders, substance misuse and violent crime arrest were not consistent with a causal interpretation. We report two principal findings.

First, we estimated that for each increase of $15 000 in family income measured at age 15 years, the offspring were at least 9% less likely to have been diagnosed with any of the examined psychiatric disorders, engaged in substance misuse or been arrested for a violent crime. Consistently with some previous research that has not used family-based designs,^3–5^^,^^33^ we found that these associations remained but were attenuated by approximately half following adjustments for socio-demographic factors and parental history of psychiatric disorders, substance misuse and violent crime arrest. Second, we found that all of the associations were no longer present once unmeasured familial confounders shared by siblings were accounted for. This was done by comparing rates of the outcomes between siblings of different ages who grew up in the same household during periods when family income levels varied. The lack of within-family associations could not be explained by insufficient income variation within families or alternative definitions of exposures and outcomes. We found that the results for siblings were generalizable to the population across all of the outcomes and our complementary cousin-comparison analyses provided additional support to our conclusions, as the results were commensurate across vastly different model assumptions. Together, the findings therefore support the hypothesis that the same underlying risk factors that have led to low family income are those that increase the risks of the outcomes of interest.

Our findings are in keeping with an earlier Swedish nationwide study that reported full confounding of the population-wide associations between childhood family income and subsequent risks for violent crime convictions and substance misuse in sibling-comparison models.^34^ However, this previous study did not examine the other more common outcomes and was limited by having data on only five birth cohorts. Our findings are also consistent with large-scale within-individual studies that have demonstrated that the associations between income and risks for psychiatric morbidity and violent perpetration^35^^,^^36^ in adults appear to be weak, if present at all, when controlled for unmeasured confounders.^37–39^ Studies that have examined the associations between family income and less severe parent-reported offspring behavioural problems in pre-adolescent children using within-family designs have nevertheless been suggestive of causal effects but the evidence remains weak.^40–43^ These investigations have typically been statistically underpowered due to small sample sizes and complex imputation techniques have been required to handle considerable attrition rates across all sample sizes. Importantly, they have typically reported marginal within-family associations that were sharply reduced relative to corresponding between-family associations. Furthermore, even if the small reported effects in these previous studies are validated, they could nevertheless be explained by residual genetic confounding, as biological full siblings only share about half of their co-segregating genes.

An important implication of our findings, if replicated in other contexts, is that interventions that primarily focus on improving parental earnings will unlikely lead to reductions in the rates of psychiatric disorders, substance misuse and violent crime arrests in their offspring. It remains possible, however, that such interventions may have a positive impact on outcomes that we have not considered. Whereas low family income may potentially be helpful in identifying families at risk, the specific interventions should target factors that have been found to predict the outcomes of interest in large-scale studies with proper controls for unmeasured confounding or have been subject to testing in adequately sized trials. We note that family income will unlikely mediate such associations, as within-family differences in income, regardless of their causes, were not associated with any of the outcomes of interest.

Our results further underline that observational studies with adjustments for only measured confounders should be cautiously interpreted. We initially found that sizable family income differences persisted when we accounted for a wide range of measures of parental psychiatric disorders, substance misuse and violent crime arrests. Such measures are commonly used in the literature but their ability to account for familial risk remains limited because they typically (i) only account for a small number of relatives, (ii) assume that the relatives have equivalent risks of meeting criteria for the outcomes and (iii) ignore subclinical symptoms by their reliance on clinical diagnoses.^44^^,^^45^ By adopting the sibling-comparison approach, we were able to obtain more accurate estimates of the associations because we accounted for a more comprehensive set of shared familial vulnerabilities.

Our study has a number of strengths. The nationwide Finnish registries allowed us to study >650 000 individuals and 185 000 families with minimal selection biases. Several relative and absolute measures of family income were derived from high-quality population registers, which provided detailed accounts of the family-income trajectories across the childhood and adolescence of the participants. We used data on clinically ascertained diagnoses and police arrests to identify individuals with psychiatric disorders in addition to those who engaged in substance misuse and violent crime. Importantly, we were able to account for unmeasured familial confounding by adopting the sibling-comparison design.

Some limitations should be noted. First, the patient data were restricted to secondary care settings only. However, we obtained similar results when we examined psychotropic drug prescriptions (e.g. antipsychotics and mood stabilizers, antidepressants and anxiolytics), which are commonly prescribed in primary care settings, as outcomes. Second, whilst the sibling-comparison design offers a powerful approach for controlling for unmeasured familial confounding, it does not inform the extent to which the familial confounding is genetic or environmental in origin because siblings share both sources of influences. However, etiological studies of income,^11^ violent crime convictions^46^ and psychiatric disorders^15^^,^^16^^,^^18^ have typically reported modest contributions of shared environmental influences. Elucidating the relative contributions of genetic and environmental factors to the confounding is an important future research question that could be addressed using quantitative genetic models.^10^^,^^47^ Third, the analyses of violent crime perpetration were based on arrest data, which had the benefit of including violent perpetrators who were not convicted but at the cost of including individuals who were subsequently acquitted. Our findings did, however, replicate an earlier nationwide Swedish study that specifically investigated violent crime convictions as the outcome.^34^ Fourth, the generalizability of our findings remains unclear. Although rates of psychiatric disorders^48^ and assaults^49^ are similar across Western Europe, there are smaller income differences in Finland than in other high-income countries.^1^ However, causality cannot be inferred solely based on the magnitude of the income differences because selection mechanisms into different income groups vary between countries and should be carefully modelled for before conclusions are drawn. Future studies may therefore consider using large-scale, representative and genetically informative data from non-Nordic countries to test for cross-country differences. As randomized controlled trials of antipoverty programmes and cash-transfer interventions have shown some promise in low-income countries,^50^ large-scale experimental studies with long follow-ups are warranted to clarify whether the effects persist over time and, importantly, whether they have an impact on adulthood outcomes in the offspring of the recipients. It is possible that these mechanisms are different between low-income and high-income countries.

In summary, we found that previously widely reported associations between family income and subsequent risks of being diagnosed with psychiatric disorders, engaging in substance misuse and being arrested for a violent crime were accounted for by unmeasured familial factors. Our findings support a selection rather than causation hypothesis, where underlying familial risk factors that increase the likelihood of psychiatric disorders, substance misuse and violent crime arrest overlap with those explaining family-income differences. Interventions that aim to prevent these outcomes in low-income families could therefore consider other modifiable factors where causality can be demonstrated.

## Supplementary data

Supplementary data are available at *IJE* online.

## Ethics approval

We were granted permission to use anonymized data from Statistics Finland following an approval by their Ethics Board (TK-53–525-11). Informed consent is not a requirement for register-based studies in Finland.

## Funding

The study was supported by the Wellcome Trust Senior Research Fellowship to S.F. [#202836/Z/16/Z] and the Academy of Finland [#308247, #294861, #316595]. The funders were not involved in the design and conduct of the study; collection, management, analysis and interpretation of the data; or preparation, review or approval of the manuscript.

## Data availability

Finnish privacy laws prohibit us from making individual-level data publicly available. Aggregate data are provided in the paper. Researchers who are interested in replicating our work using individual-level data can seek access via Statistics Finland. For more information, see https://www.stat.fi/tup/mikroaineistot/index_en.html.

## Author contributions

A.S. and S.F. contributed to the conception and design of the study, A.S. conducted the statistical analyses and with S.F. drafted the paper. All authors critically revised the draft and approved the final version.

## Conflicts of interest

None declared.

## Supplementary Material

dyab099_Supplementary_Data
