# Supplementary material for: No causal associations between childhood family income and subsequent psychiatric disorders, substance misuse and violent crime arrests: a nationwide Finnish study of >650 000 individuals and their siblings
Source: Int J Epidemiol. 2021 May 29;50(5):1628–38. doi: 10.1093/ije/dyab099 (PMC8580272; doi:10.1093/ije/dyab099)
Supplement: dyab099_Supplementary_Data [file dyab099_supplementary_data.docx]

**Supplementary Table S1. ICD codes**

|  | **ICD-8-FI**  **(1969-1986)** | **ICD-9-FI**  **(1987-1995)** | **ICD-10-FI**  **(1996-)** |
| --- | --- | --- | --- |
| Severe mental illness | 295-299 [excl. 296.2] | 295-299 [excl. 296.1A-296.1D, 296.1F-296.1G] | F20-F31 |
| Schizophrenia | 295 | 295 | F20 |
| Schizophrenia-spectrum disorder | 295, 297-299 | 295, 297-298 | F20-F29 |
| Bipolar disorder | 296 [excl. 296.2] | 296 [excl. 296.1A-296.1D, 296.1F-296.1G] | F30-F31 |
| Depression | 296.2. 300.4 | 296.1 [excl. 296.1E], 296.8A, 300.4A | F32-F39  [excl. F32.3 and F33.3] |
| Anxiety | 300 [excl. 300.4] | 300 [excl. 300.4] | F40-F42 [excl. F41.2], F44-F45, F48 |
| Substance use disorder | 291-292, 303-304 | 291-292, 303-304,  305 [excl. *.1] | F10-F12, F14-F16, F19 [excl. F1*.5] |
| Alcohol use disorder | 291, 303 | 291, 303, 305.0 | F10 [excl. F10.5] |
| Drug use disorder | 292, 304 | 292, 304, 305.2-305.9 | F11, F12, F14-F16, F19 [excl. F1*.5] |

**Supplementary Table S2. Penal codes**

| **Penal code** | **Offence** |
| --- | --- |
|  |  |
| Violent crime |  |
| 121 | Robbery |
| 122 | Aggravated robbery, preparation of aggravated robbery |
| 123 | Extortion, aggravated extortion |
| 201 | Manslaughter |
| 202 | Murder |
| 203 | Attempted manslaughter, murder or killing |
| 204 | Infanticide |
| 205 | Killing |
| 206 | Preparation of aggravated crime against life or health |
| 211 | Assault |
| 212 | Aggravated assault |
| 213 | Petty assault |
| 221 | Negligent homicide, grossly negligent homicide |
| 222 | Negligent injury, grossly negligent injury |
| 223 | Other crimes against life and health |
| 231 | Sexual abuse of a child, aggravated sexual abuse of a child |
| 232 | Rape, aggravated rape, rape |
| 233 | Other sexual crimes |
| 301 | (Violent) resisting of an official in the performance of his duties |
|  |  |
| Drug-related crime |  |
| 451 | Narcotics offence |
| 452 | Aggravated narcotics offence |
| 456 | Preparation of a narcotics offence |
| 457 | Abetment of a narcotics offence, abetment of an aggravated narcotics offence |
| 458 | Narcotics abuse offence |

**Supplementary Table S3. ATC codes**

| **Medication** | **ATC code** |
| --- | --- |
| Antipsychotics | N05A (excl. N05AN01) |
| Mood stabilizers | N03AF01, N03AF02, N03AG01,  N03AX09, N05AN01 |
| Antidepressants | N06A |
| Anxiolytics | N05B |
| Medications used in alcohol and opioid addiction treatment | N07BB and N07BC |

**Supplementary Table S4. Measured confounders**

| **Measured confounder** | **Definition** |
| --- | --- |
| Sex | 0=Male; 1=Female |
| Birth year | Categorical measure with 11 levels (1986-1996). |
| Birth order | Categorical measure with 4 levels based on the order of birth within each mother: 1=First-born, 2= second-born, 3=third-born, 4=fourth-born or higher. |
| Immigrant background | 0=Both parents were born in Finland; 1=At least one parent was born abroad, |
| Urbanicity | Statistics Finland’s classification of the level of urbanicity across municipalities in three levels (1=urban municipalities, 2=semi-urban municipalities and 3=rural municipalities). Measured at age 1. |
| Single parent household | Family type at birth either single mother or father with at least one child. Measured at age 1. |
| Parental educational attainment | The highest level of education achieved by either parent in three categories: 1=primary level (International Standard Classification of Education [ISCED] 2011 levels: 0-2), 2=secondary level (ISCED 2011 levels: 3-5) or 3=tertiary level (ISCED 2011 levels: 6-8). |
| Parental psychiatric disorders and antisocial behaviors | We used the same definitions as the outcomes measured in the offspring. |

**Supplementary Figure S1. Associations between family income at age 15 years (in units of $15,000) and subsequent psychiatric disorders, substance misuse and violent crime arrest excluding incomplete outpatient care data prior to 2006 among individuals born in Finland 1986-1996 and followed up until 31 December 2017 (31 December 2018 for substance misuse)**

**
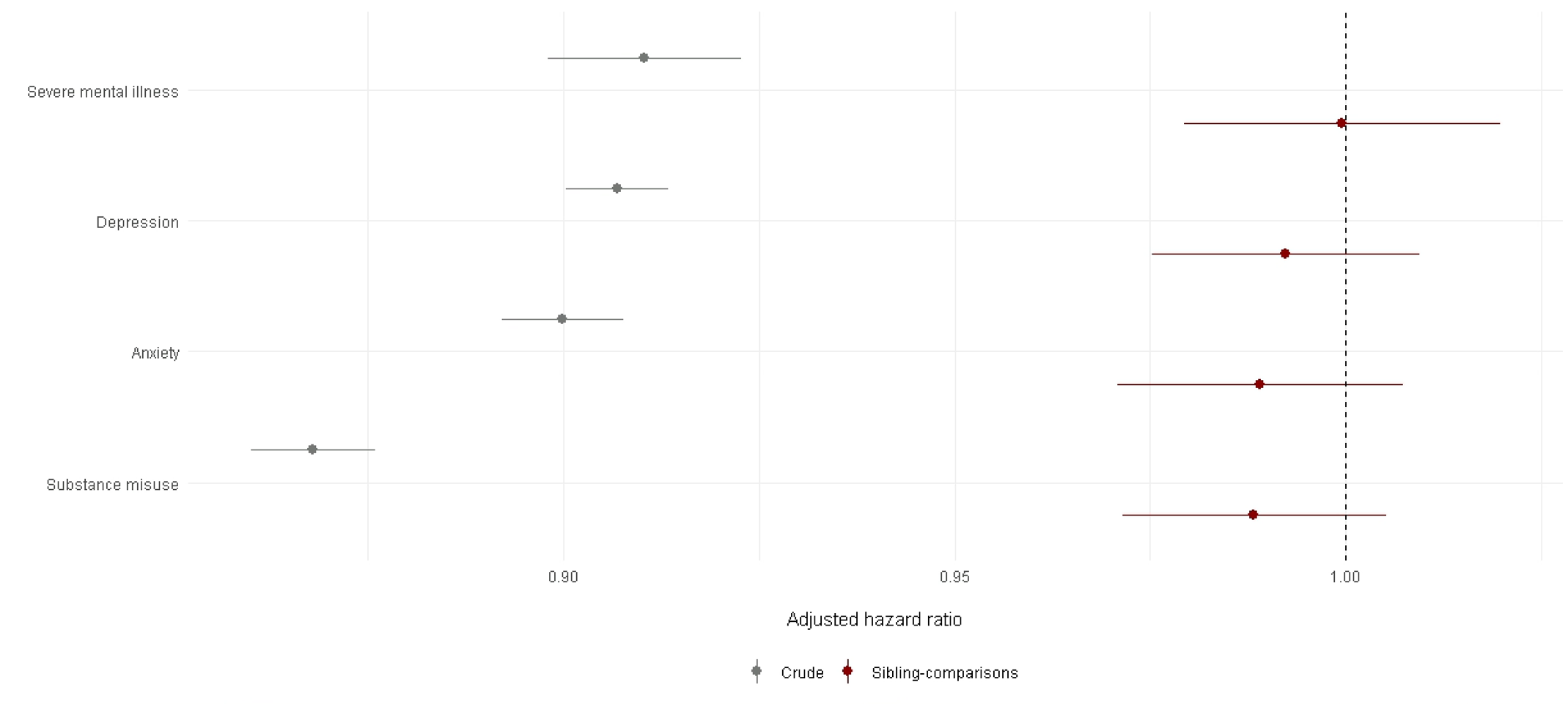
**

*Notes: All models were adjusted for sex, birth year and birth order.*

**Supplementary Figure S2. Associations between family income at age 15 years (in units of $15,000) and subsequent psychiatric disorders, substance misuse and violent crime arrest excluding individuals who met criteria for the outcomes prior to the baseline of the study among individuals born in Finland 1986-1996 and followed up until 31 December 2017 (31 December 2018 for substance misuse)**

**
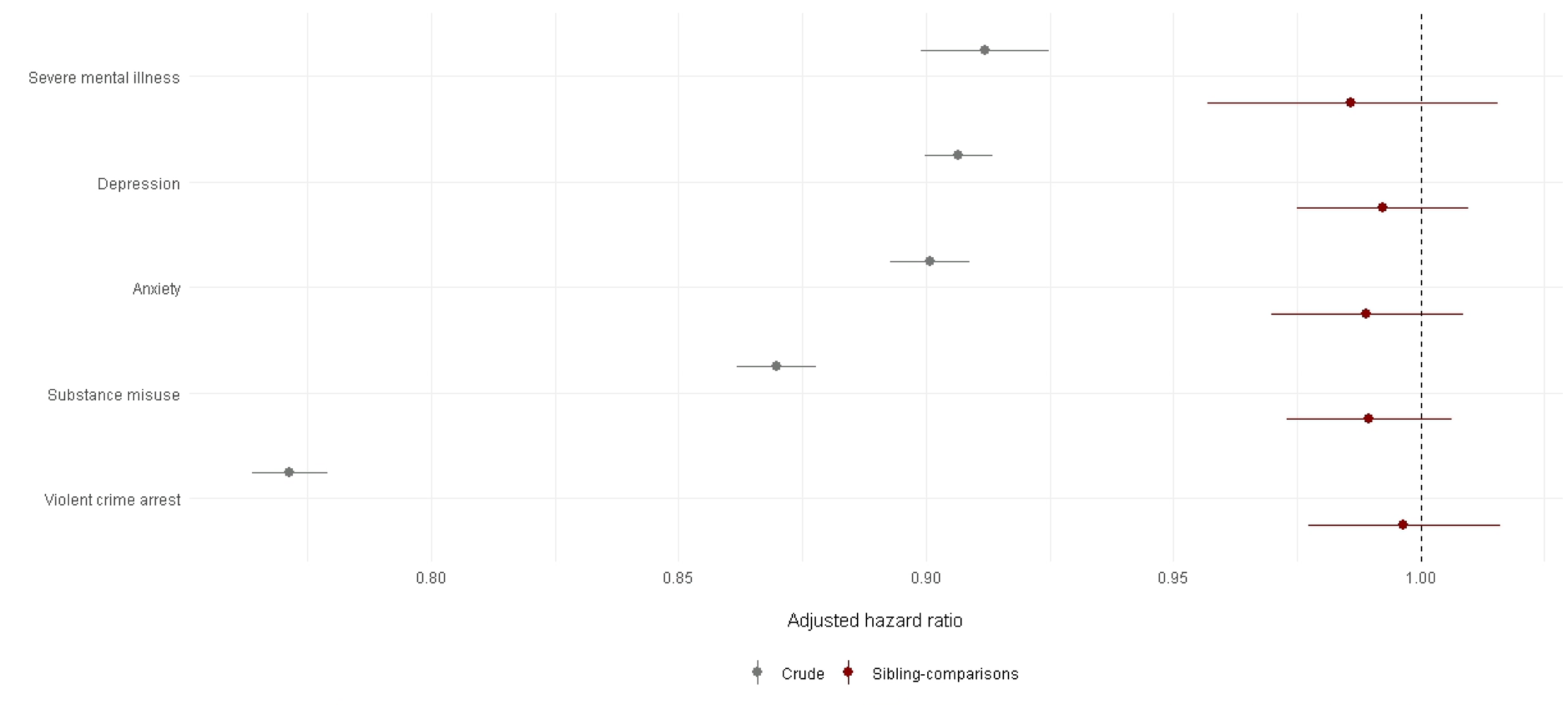
**

**Supplementary Figure S3. Associations between family income at age 15 years and subsequent specific severe mental illnesses, substance misuse and psychotropic medications among individuals born in Finland 1986-1996 and followed up until 31 December 2017 (31 December 2018 for the prescription drug outcomes)**

**
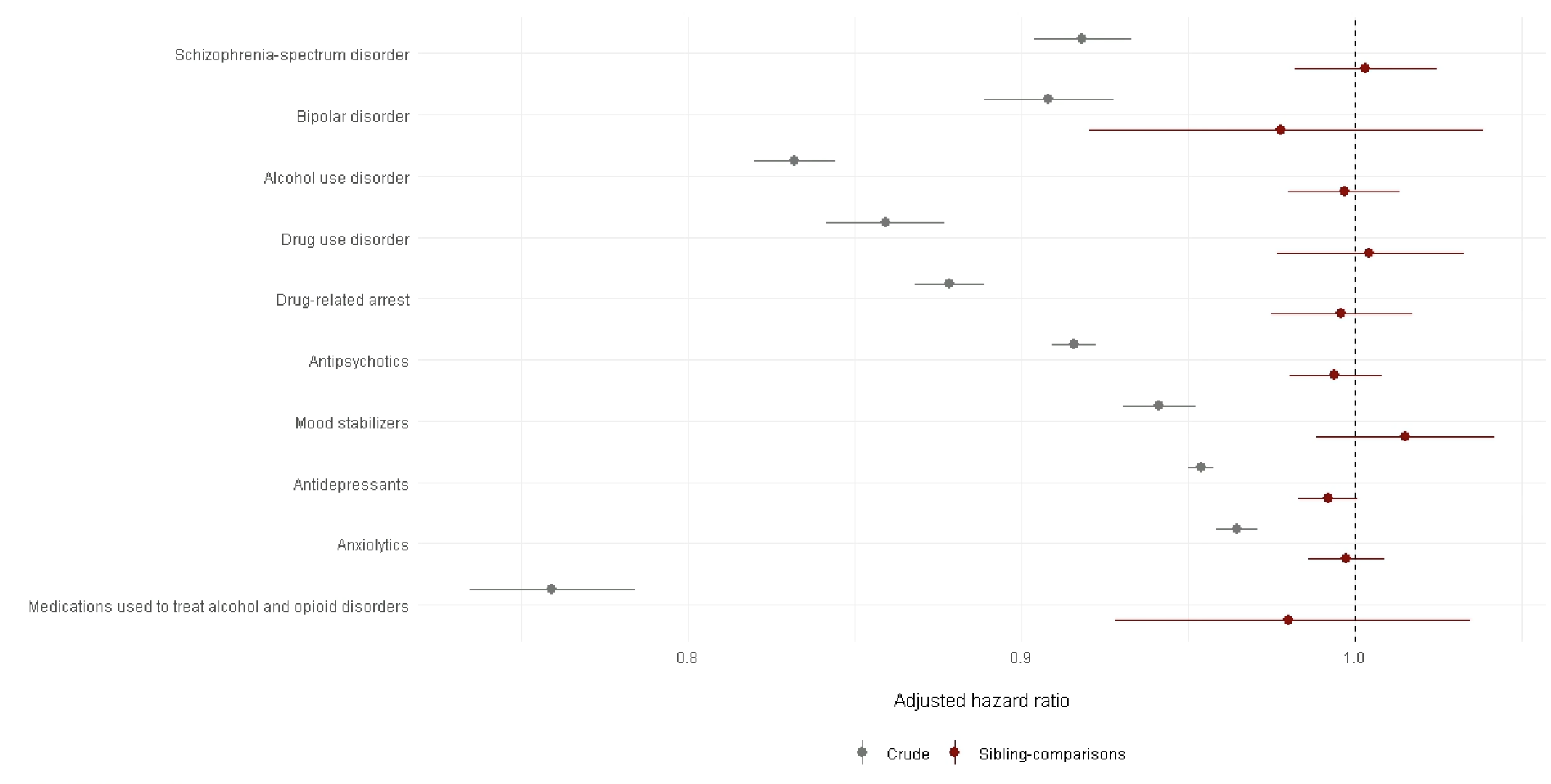
**

*Notes: All models were adjusted for sex, birth year and birth order.*

**Supplementary Figure S4. Associations between gross and disposable family income, with and without corrections for family size, at age 15 years (in units of $15,000 [USD] or €15,000) and subsequent psychiatric disorders, substance misuse and violent crime arrest among individuals born in Finland 1986-1996 and followed up until 31 December 2017 (31 December 2018 for substance misuse)**

**
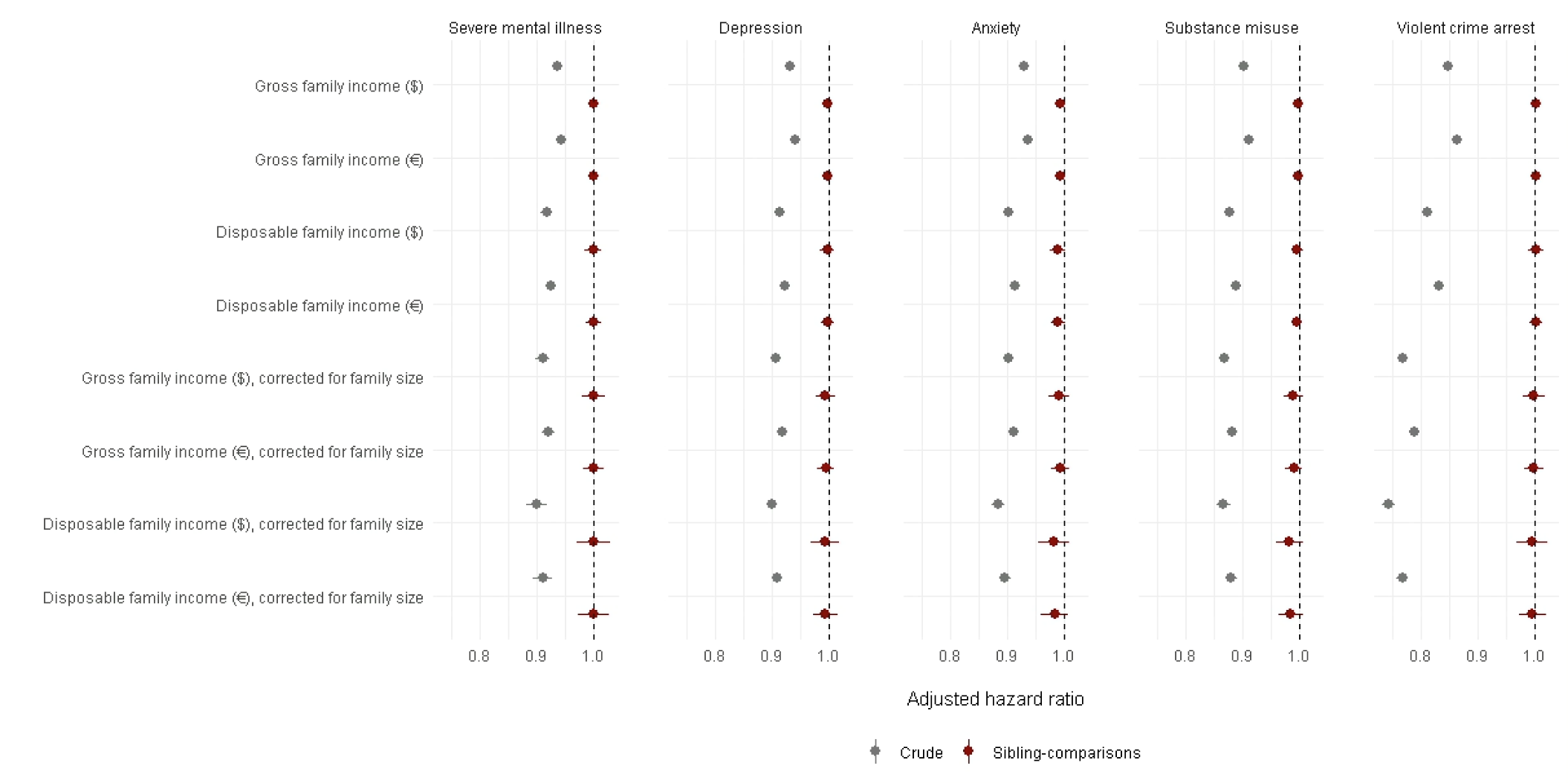
**

*Notes: All models were adjusted for sex, birth year and birth order.*

**Supplementary Figure S5. Associations between alternative indicators of low parental socioeconomic status (e.g., receipt of means-tested social assistance, unemployment and disability pension benefits) at age 15 years and subsequent psychiatric disorders, substance misuse and violent crime arrest among individuals born in Finland 1986-1996 and followed up until 31 December 2017 (31 December 2018 for substance misuse)**

*
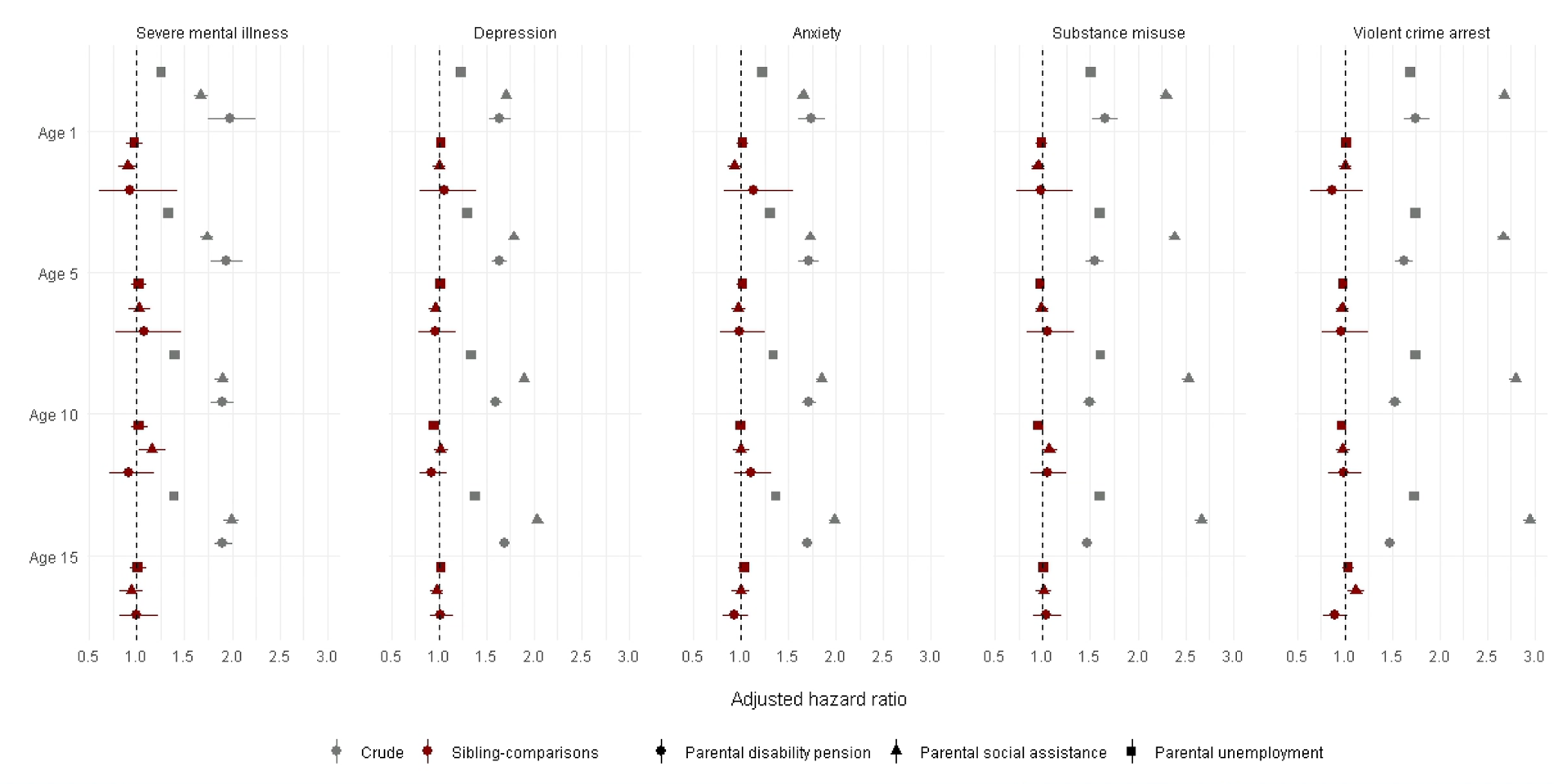
*

*Notes: All models were adjusted for sex, birth year and birth order. None of the sibling estimates survived multiple testing correction (P>0.05).*

**Supplementary Figure S6. Crude associations between family income at age 15 years and subsequent psychiatric disorders, substance misuse and violent crime arrest in the full sample versus in the siblings only subset among individuals born in Finland 1986-1996 and followed up until 31 December 2017 (31 December 2018 for substance misuse)**

**
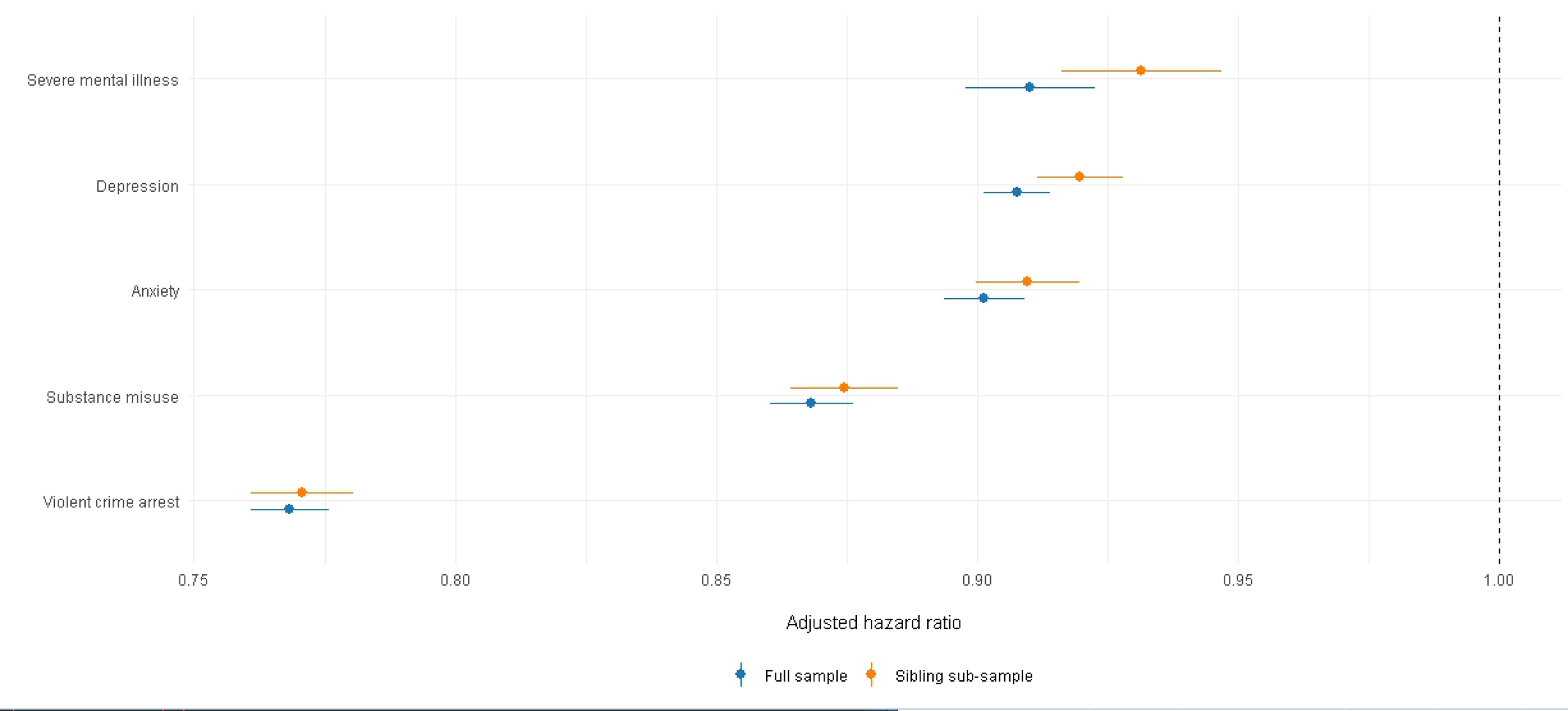
**

*Notes: All models were adjusted for sex, birth year and birth order.*

**Supplementary Figure S7. Within-extended family associations between family income at age 15 years and subsequent psychiatric disorders, substance misuse and violent crime arrest, stratified across all cousins and cousins who resided in different municipalities from a base sample of individuals born in Finland 1986-1996 and followed up until 31 December 2017 (31 December 2018 for substance misuse)**

*
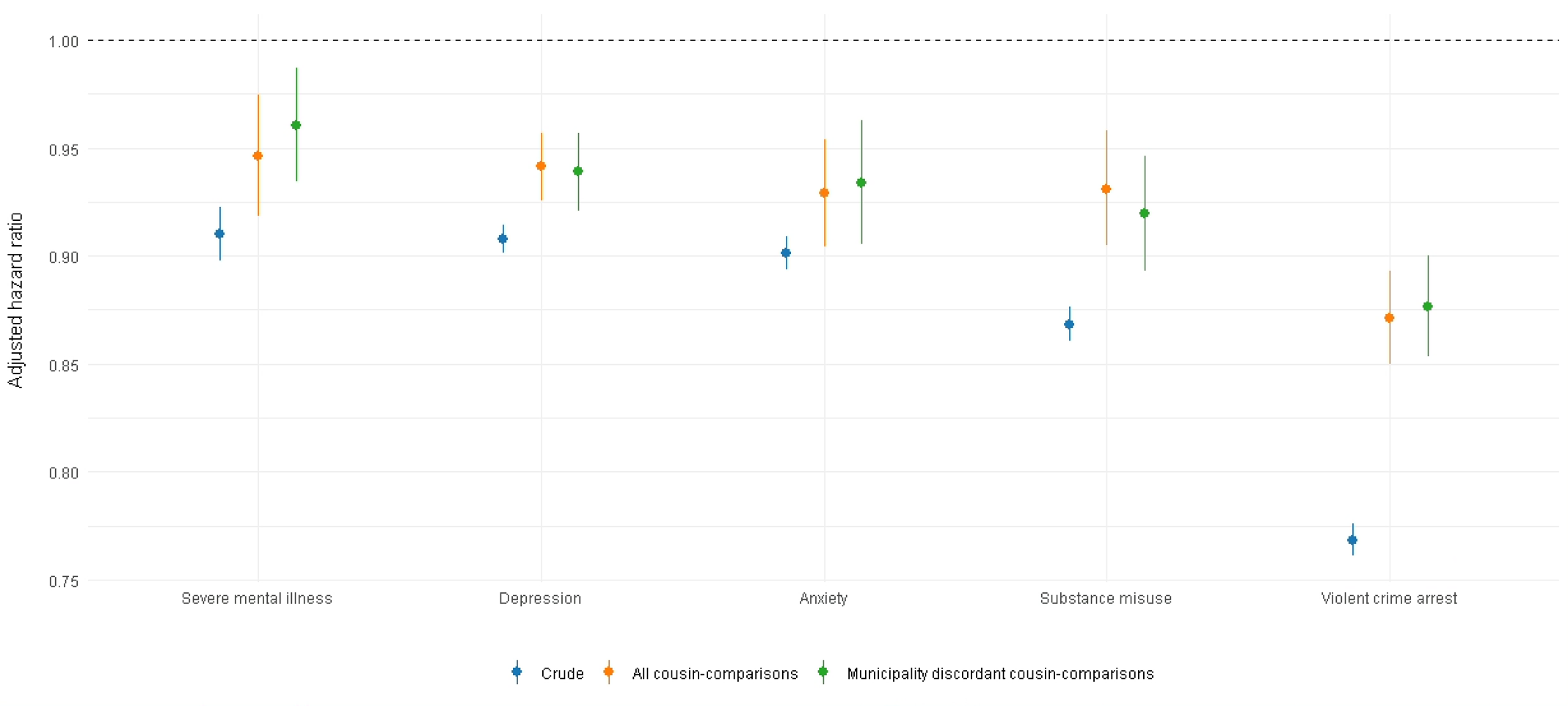
*

*Notes: All models were adjusted for sex, birth year and birth order.*

**Group-based trajectory models**

We fitted a series of group-based trajectory models (GBTMs),^1^ also known as latent class growth curve models, to estimate family income trajectories^2^ in our dataset by using the *traj* plug-in in Stata 16.1 MP.^3^ GBTMs attempt to identify finite groups of individuals who follow distinct developmental trajectories, which are captured by a categorical latent variable or *latent classes*. Conceptually, these models summarize the longitudinal heterogeneity of family income exposures into a smaller number of typical trajectories.^4^ The identification of the number of latent classes (e.g., family income trajectories) supported by the data follows an sequential process whereby researchers use fit indices and other criteria (e.g., cut-off values for group size and theoretical plausibility) to determine whether the inclusion of additional latent classes improve the model fit.^5^

Our input data consisted of annual gross family income measures (in 2019 USD values) between ages 1 to 15 years that were corrected for family size and log transformed. To facilitate model convergence, we excluded individuals who had zeros in any of the family income throughout the examined time period (*n*=3637; 0.6%). We specified the models to treat these variables as censored normal using the CNORM option and we further specified age as a third-degree polynomial across the latent classes. Akaike’s Information Criterion (AIC) and the Bayesian Information Criterion (BIC) were used to determine the number of latent classes supported by the data. As lower AIC/BIC values indicate better model fit, we found that a model with 4 latent classes provided the best fit (**Supplementary Figure S8**).

The model provides (posterior) probabilities for each individual to belong to each of the specific latent classes in order to assess its classification accuracy. In a well-fitting model, the individuals have a high probability of belonging to the latent class that the model has assigned them to. We found that out model had excellent classification accuracy with average probabilities of belonging to the assigned latent class ranging between 93% to 96% across the four latent classes. A range of 70% to 80% is typically considered to be acceptable.^6^

The latent classes, or family income trajectories, are presented in **Supplementary Figure S9**. The predicted family income levels varied considerably in their baseline means and trends across time. However, as all of the trajectories were generally increasing over time, we labeled them based on their overall level: “Lowest” (comprising of 13.6% of the sample), “Low” (44.3%), “Middle” (34.2%), and “High” (7.9%).

When re-ran the crude models with the categorical measure of family income trajectories as exposure, we found that those who were exposed to lower income trajectories had elevated risks of the outcomes (**Supplementary Figure S10**). In the subset of siblings (*n*=424,776) that were included in these analyses, we found that 20.5% (n=87,145) were assigned to a different family income trajectory than at least one of their siblings. The population-wide associations were nevertheless fully attenuated when we used differentially exposed siblings as comparators (**Supplementary Figure S10**), thus replicating the main finding of the paper, which suggests that the associations are entirely confounded by unmeasured familial risks.

**References**

1. Jones BL, Nagin DS. Advances in Group-Based Trajectory Modeling and an SAS Procedure for Estimating Them. *Sociol Methods Res*. 2007;35(4):542-571.

2. Björkenstam E, Cheng S, Burström B, Pebley AR, Björkenstam C, Kosidou K. Association between income trajectories in childhood and psychiatric disorder: a Swedish population-based study. *J Epidemiol Community Health*. 2017;71(7):648-654.

3. Jones BL, Nagin DS. A note on a Stata plugin for estimating group-based trajectory models. *Sociol Methods Res*. 2013;42(4):608-613.

4. Herle M, Micali N, Abdulkadir M, et al. Identifying typical trajectories in longitudinal data: modelling strategies and interpretations. *Eur J Epidemiol*. 2020;35(3):205-222.

5. Nylund KL, Asparouhov T, Muthén BO. Deciding on the number of classes in latent class analysis and growth mixture modeling: A Monte Carlo simulation study. *Struct Equ Model*. 2007;14(4):535-569. doi:10.1080/10705510701575396

6. Cote S, Tremblay RE, Nagin D, Zoccolillo M, Vitaro F. The development of impulsivity, fearfulness, and helpfulness during childhood: patterns of consistency and change in the trajectories of boys and girls. *J Child Psychol Psychiatry*. 2002;43(5):609-618.

**Supplementary Figure S8. Model fit indices for the group-based trajectory models**


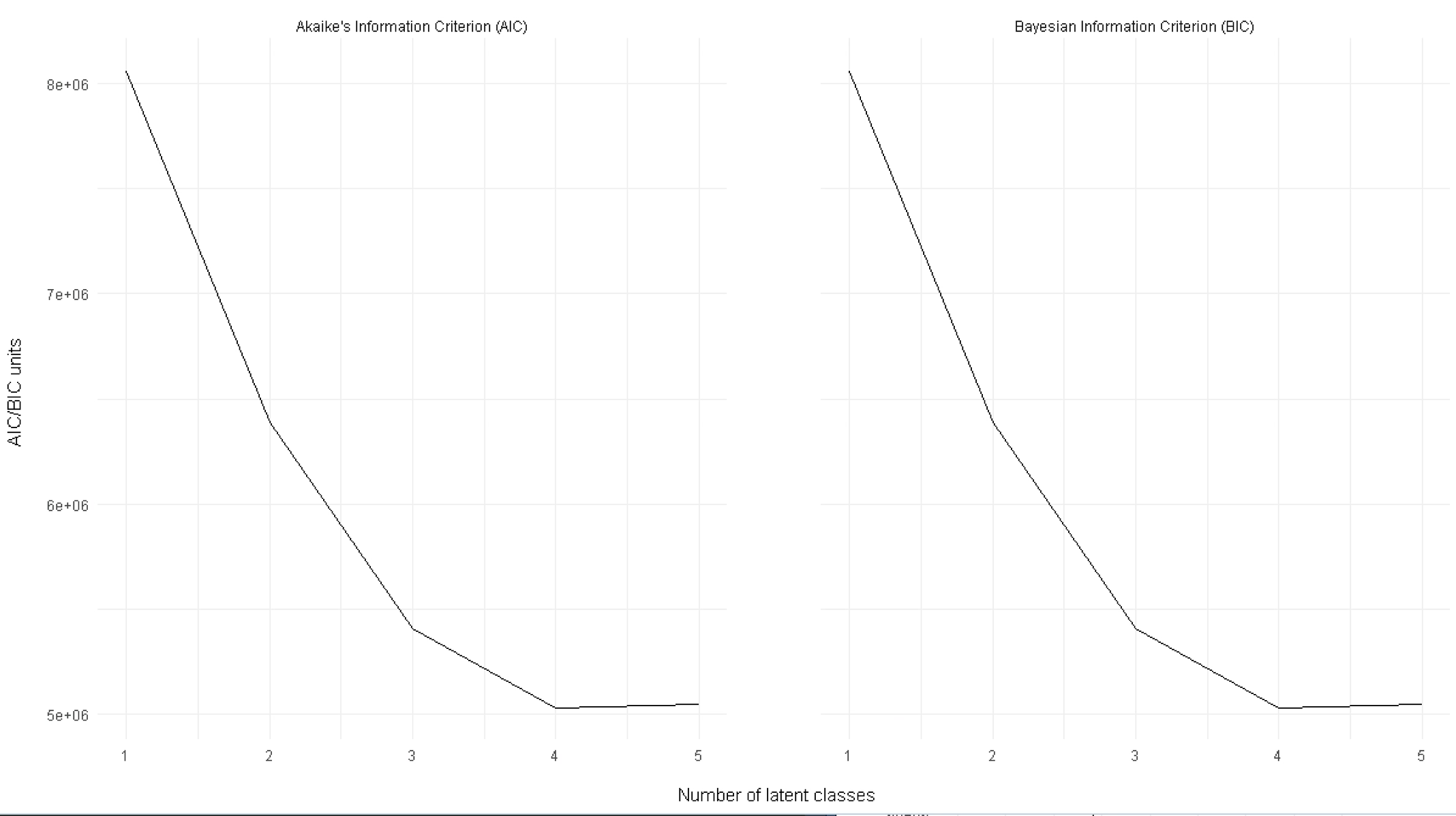


**Supplementary Figure S9. Family income trajectories measured between ages 1 to 15 years among individuals born in Finland 1986-1996. The income measures were corrected for family size and log transformed.**

**
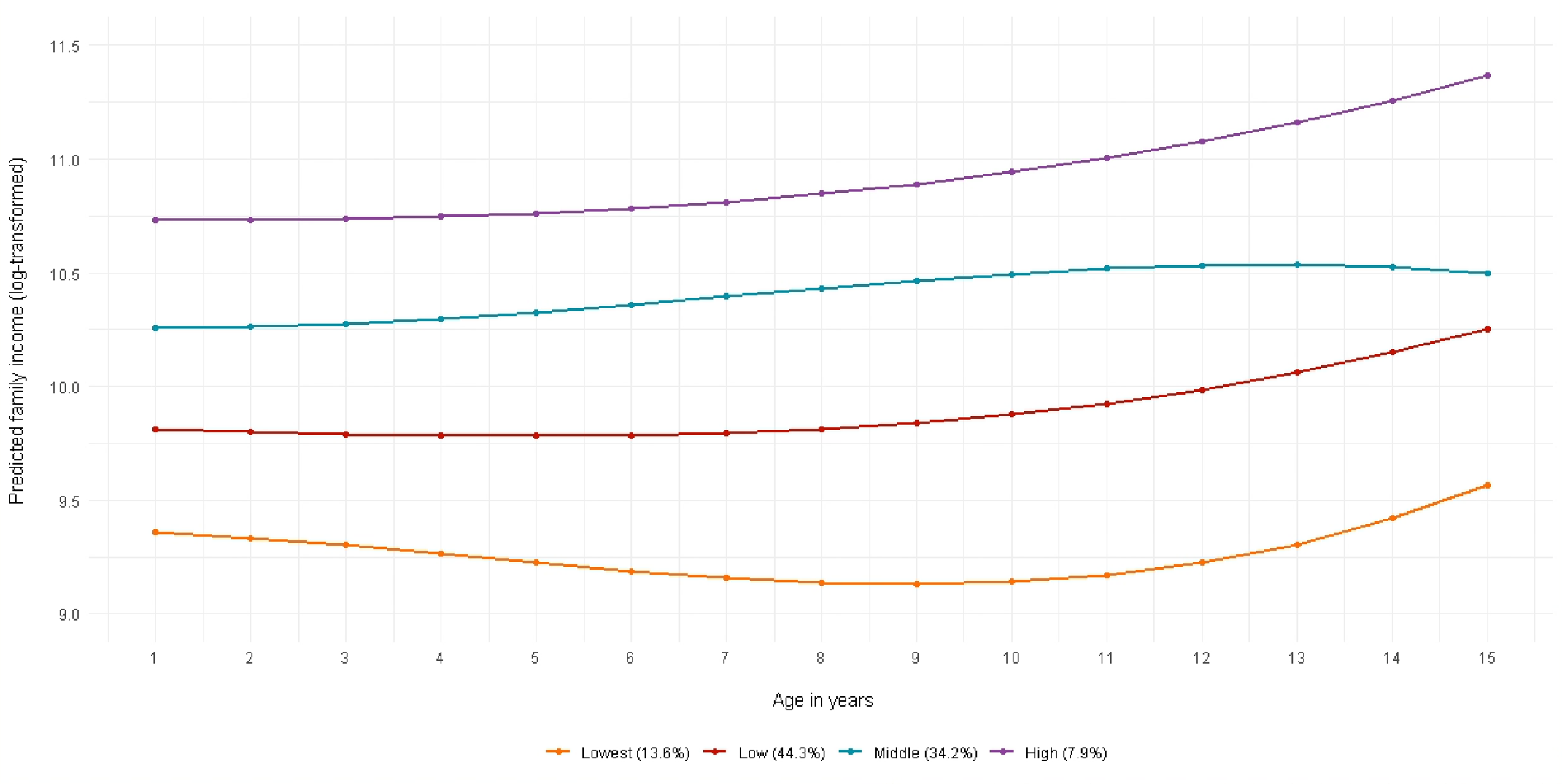
**

**Supplementary Figure S10. Associations between family income trajectories and subsequent psychiatric disorders, substance misuse and violent crime arrest (“Low” family income trajectory as the reference category) among individuals born in Finland 1986-1996 and followed up until 31 December 2017 (31 December 2018 for substance misuse)**


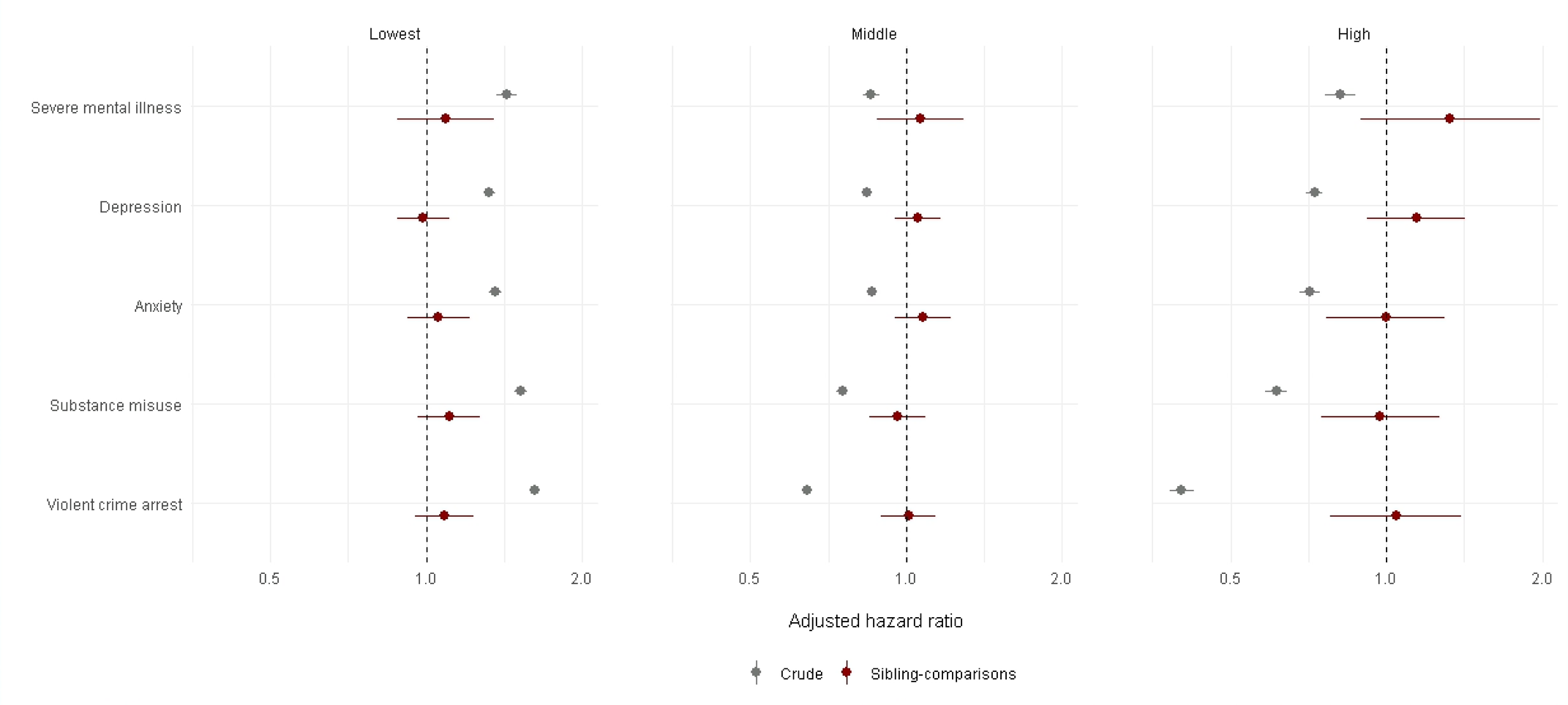


*Notes: All models were adjusted for sex, birth year and birth order.*
